# A dynamic discount pricing strategy for viral marketing

**DOI:** 10.1371/journal.pone.0208738

**Published:** 2018-12-28

**Authors:** Xiang Zhong, Juan Zhao, Lu-Xing Yang, Xiaofan Yang, Yingbo Wu, Yuan Yan Tang

**Affiliations:** 1 School of Big Data & Software Engineering, Chongqing University, Chongqing, 400044, China; 2 School of Information Technology, Deakin University, Melbourne, VIC 3125, Australia; 3 Faculty of Science and Technology, UOW College Hong Kong/Community College of City University, Kowloon, Hong Kong; 4 Department of Computer and Infomation Science, The University of Macau, Macau, China; Monash University, AUSTRALIA

## Abstract

Viral marketing has been one of the main marketing modes. However, theoretical study of viral marketing is still lacking. This paper focuses on the problem of developing a cost-effective dynamic discount pricing strategy for a viral marketing campaign. First, based on a novel word-of-mouth propagation model, we model the original problem as an optimal control problem. Second, we show that the optimal control problem admits an optimal control and present the optimality system for solving the optimal control problem. Next, we solve some optimal control models to get their respective optimal dynamic discount pricing strategies. Finally, we examine the effect of some factors on the maximum marketing profit. These results contribute to gaining insight into viral marketing.

## 1 Introduction

Viral marketing, also known as word-of-mouth (WOM) marketing, is an effective marketing mode, in which the marketing information spreads in the form of WOM among customers [1]. When consumers willingly become promoters of a product or service and spread the word to their friends, they are driven to do so either through an explicit incentive or simply out of a desire to share the product benefits with friends [2]. With the proliferation of online social networks, viral marketing can achieve market adoption more cost-effectively than traditional marketing modes such as TV advertising [3–6].

To accurately evaluate the cost profit of a viral marketing campaign, we have to gain a deep insight into the laws of WOM propagation [7]. For this purpose, in recent years some WOM propagation models based on homogeneous networks have been proposed [8–13]. Yet, it was reported that many online social networks are highly heterogeneous and highly structured [14–16]. Consequently, WOM propagation models based on heterogeneous networks have received considerable interest [17–20]. However, all of these work except [20] were done through simulation experiments, not shaping a theoretic system. To establish a general theoretic framework about viral marketing, we need to introduce and study WOM propagation models based on arbitrary networks.

Node-level epidemic modeling is recognized as an effective approach to the understanding of complex propagation phenomena over arbitrary networks [21]. In a node-level epidemic model, the probability of each node in any given state obeys a separate differential equation. As a result, the effect of the network structure on the epidemic process is accounted for [22]. In recent years, the node-level epidemic modeling technique has been applied to areas as diverse as malware spreading [23–28], rumor spreading [29, 30], and cyber defense [31–33]. To our knowledge, this epidemic modeling technique has not been employed to characterize the propagation of WOM over arbitrary networks.

Discount is one of the major marketing tools [6]. This paper focuses on the dynamic discount pricing (DDP) problem, i.e., the problem of developing a cost-effective dynamic discount pricing (DDP) strategy for a viral marketing campaign. First, we propose a node-level WOM propagation model with discount mechanism. On this basis, we model the DDP problem as an optimal control problem we refer to as the DDP model problem. Second, we show that the DDP model problem admits an optimal control, and we derive the optimality system for solving the DDP model problem. Next, we solve some DDP models to get the corresponding optimal DDP strategies. Finally, we examine the effect of some factors on the maximum marketing profit. These results contribute to the deep understanding of viral marketing.

The subsequent materials are organized in this fashion: Section 2 models the DDP problem as the DDP model problem. Sections 3 and 4 develop a method for solving the DDP model problem and use the method to solve some DDP models, respectively. The influence of some factors on the maximum marketing profit is examined in Section 5. Section 6 summarizes this work.

## 2 The modeling of the dynamic discount pricing problem

This section focuses on the following problem.

*Dynamic discount pricing (DDP) problem*: For a marketing campaign launched by a merchant, develop a dynamic discount pricing strategy to maximize the profit of the merchant.

For this purpose, this section is devoted to the modeling of the DDP problem according to the four-step procedure: (1) introduce basic terminologies and notations, (2) formulate dynamic discount pricing strategies, (3) establish a WOM propagation model, and (4) model the DDP problem as an optimal control problem.

### 2.1 Basic terminologies and notations

Suppose a merchant intends to launch a viral marketing campaign in the time horizon [0, *T*]. Let *V* = {1, 2, …, *N*} denote the target market for the campaign, i.e., the set of all customers and potential customers in the campaign. For brevity, we refer to all customers and potential customers as nodes. Define the *influence network* of the target market as a network *G* = (*V*, *E*), where (*i*, *j*) ∈ *E* represents that node *i* has a direct influence on node *j* through online social networks (OSNs). The merchant can have full knowledge of the influence network by means of an OSN analysis software. Let **A** = (*a*_*ij*_)_*N* × *N*_ denote the adjacency matrix of *G*, i.e, *a*_*ij*_ = 1 or 0 according as (*i*, *j*) ∈ *E* or not.

Generally speaking, every node in the influence network has a certain influence in the marketing campaign, and a node with a larger out-degree has a larger influence [34]. In this paper, we take the normalized quantity

$$\begin{array}{c} d_{i}=\frac{\sum_{j=1}^{N}a_{ij}}{\max_{1\leq k\leq N}\sum_{j=1}^{N}a_{kj}} \end{array}$$ (1)

as the measure of the influence of node *i*.

### 2.2 Dynamic discount pricing strategies

Suppose for sales promotion, the merchant decides to give to each node a certain discount, and the discount rate given to each node is proportional to his or her influence. Let *θ*(*t*) denote the basic discount rate at time *t*. Then the discount rate given to node *i* at time *t* is *d*_*i*_ *θ*(*t*).

We refer to the function *θ* defined by *θ*(*t*), 0 ≤ *t* ≤ *T*, as a *dynamic discount pricing (DDP) strategy*. For technical reasons, we assume *θ* is both Lebesgue integrable and Lebesgue square integrable [35]. That is, the admissible set of DDP strategies is

$$\begin{array}{c} \Theta =\{\theta \in L\left[0,T\right]\cap L^{2}\left[0,T\right]|0\leq \theta \left(t\right)\leq 1,0\leq t\leq T\}, \end{array}$$ (2)

where *L*[0, *T*] represents the set of all Lebesgue integrable functions defined on [0, *T*], *L*^2^[0, *T*] represents the set of all Lebesgue square integrable functions defined on [0, *T*].

### 2.3 A WOM propagation model

Suppose each and every node in the target market is in one of four possible states: *susceptible*, *infected*, *positive*, and *negative*. Susceptible nodes are those who currently have intentions to purchase new items. Infected nodes are those who currently have no intentions to purchase new items, but have previously purchased some items and have made no comment on the items. Positive nodes are those who currently have no intentions to purchase new items, but have previously purchased some items and have made a general positive comment on the items. Negative nodes are those who have no intentions to purchase new items, but have previously purchased some items and have made a general negative comment on the items. Initially, all nodes are susceptible.

Let *X*_*i*_(*t*) = 0, 1, 2, and 3 denote that node *i* is susceptible, infected, positive, and negative at time *t*, respectively. Then the vector

$$\begin{array}{c} X\left(t\right)=(X_{1}\left(t\right),\cdots ,X_{N}\left(t\right)) \end{array}$$ (3)

represents the state of the target market at time *t*. In particular, we have **X**(0) = **0**.

Let *S*_*i*_(*t*), *I*_*i*_(*t*), *P*_*i*_(*t*), and *N*_*i*_(*t*) denote the probabilities of node *i* being susceptible, infected, positive, and negative at time *t*, respectively.

$$\begin{array}{c} \begin{array}{c} S_{i}\left(t\right)=\mathrm{Pr}\left\{X_{i}\left(t\right)=0\right\},\;I_{i}\left(t\right)=\mathrm{Pr}\left\{X_{i}\left(t\right)=1\right\},\\ P_{i}\left(t\right)=\mathrm{Pr}\left\{X_{i}\left(t\right)=2\right\},\;N_{i}\left(t\right)=\mathrm{Pr}\left\{X_{i}\left(t\right)=3\right\}. \end{array} \end{array}$$ (4)

As *S*_*i*_(*t*) = 1 − *I*_*i*_(*t*) − *P*_*i*_(*t*) − *N*_*i*_(*t*), the vector

$$\begin{array}{c} x\left(t\right)=(I_{1}\left(t\right),\cdots ,I_{N}\left(t\right),P_{1}\left(t\right),\cdots ,P_{N}\left(t\right),N_{1}\left(t\right),\cdots ,N_{N}\left(t\right)) \end{array}$$ (5)

represents the expected state of the target market at time *t*. In particular, we have **x**(0) = **0**.

Next, let us introduce a set of hypotheses as follows.

(H_1_) Encouraged by positive comments, the susceptible node *i* purchases new items and hence becomes infected at time *t* at the rate of $\beta_{P}\sum_{j=1}^{N}a_{ji}P_{j}\left(t\right)$, where *β*_*P*_ is a positive constant. We refer to *β*_*P*_ as the *positive infection force*. This hypothesis implies that a more influential node contributes more to the marketing than a less influential node.
(H_2_) Encouraged by discount, the susceptible node *i* purchases new items and hence becomes infected at time *t* at the rate of *β*_*D*_ *d*_*i*_ *θ*(*t*), where *β*_*D*_ is a positive constant. We refer to *β*_*D*_ as the *discount infection force*. This hypothesis implies that a node who can get a higher discount rate tends to purchase items.
(H_3_) Due to good feeling of recently purchased items, each infected node makes a general positive comment and hence becomes positive at the rate of *α*_*P*_, which is a positive constant. We refer to *α*_*P*_ as the *positive comment rate*.
(H_4_) Due to bad feeling of recently purchased items, each infected node makes a general negative comment and hence becomes negative at the rate of *α*_*N*_, which is a positive constant. We refer to *α*_*N*_ as the *negative comment rate*.
(H_5_) Due to the desire of online shopping, each infected node becomes susceptible at the rate of *γ*_*I*_, which is a positive constant. We refer to *γ*_*I*_ as the *neutral desire rate*.
(H_6_) Due to the desire of online shopping, each positive node becomes susceptible at the rate of *γ*_*P*_, which is a positive constant. We refer to *γ*_*P*_ as the *positive desire rate*. Obviously, *γ*_*P*_ > *γ*_*I*_.
(H_7_) Due to the desire of online shopping, each negative node becomes susceptible at the rate of *γ*_*N*_, which is a positive constant. We refer to *γ*_*N*_ as the *negative desire rate*. Obviously, *γ*_*N*_ < *γ*_*I*_.

**Remark 1**. *The merchant can estimate the seven parameters*, *β*_*P*_, *β*_*D*_, *α*_*P*_, *α*_*N*_, *γ*_*I*_, *γ*_*P*_, *and* *γ*_*N*_, *by collecting and analyzing relevant historical data*.

**Fig 1:**
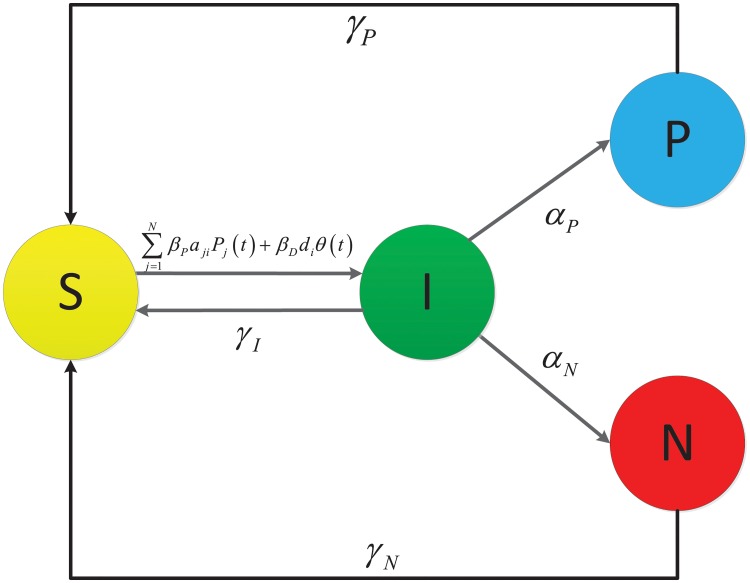
Diagram of state transitions of node *i* under the hypotheses (H_1_)-(H_7_).

These hypotheses are shown in Fig 1. So, the expected state of the target market evolves according to the following differential dynamical system:

$$\begin{array}{c} \left\{ \begin{array}{cc} \frac{dI_{i}\left(t\right)}{dt} & =[\beta_{P}\sum_{j=1}^{N}a_{ji}P_{j}\left(t\right)+\beta_{D}d_{i}\theta \left(t\right)][1-I_{i}\left(t\right)-P_{i}\left(t\right)-N_{i}\left(t\right)]\\ & -\left(\alpha_{P}+\alpha_{N}+\gamma_{I}\right)I_{i}\left(t\right),\;0\leq t\leq T,1\leq i\leq N,\\ \frac{dP_{i}\left(t\right)}{dt} & =\alpha_{P}I_{i}\left(t\right)-\gamma_{P}P_{i}\left(t\right),\;0\leq t\leq T,1\leq i\leq N,\\ \frac{dN_{i}\left(t\right)}{dt} & =\alpha_{N}I_{i}\left(t\right)-\gamma_{N}N_{i}\left(t\right),\;0\leq t\leq T,1\leq i\leq N,\\ x(0) & =0. \end{array}\right. \end{array}$$ (6)

We refer to the model as the *node-level WOM propagation model*. This model may be abbreviated as

$$\begin{array}{c} \begin{array}{cc} \frac{dx(t)}{dt} & =f(x(t),\theta (t)),\;0\leq t\leq T,\\ x(0) & =0. \end{array} \end{array}$$ (7)

### 2.4 The modeling of the DDP problem

Obviously, the gross profit of the merchant is increasing with the rate at which a susceptible node becomes infected. In this paper, we introduce an added hypothesis as follows.

(H_8_) The resulting gross profit per unit time when any susceptible node becomes infected at the rate of *β* is equal to *β* units.

The hypotheses (H_1_)-(H_2_) tell us that the susceptible node *i* becomes infected at time *t* at the rate of

$$\begin{array}{c} \beta_{P}\sum_{j=1}^{N}a_{ji}P_{j}\left(t\right)+\beta_{D}d_{i}\theta \left(t\right). \end{array}$$ (8)

In view of the hypothesis (H_8_) and the discount rate, the net profit in the infinitesimal time interval [*t*, *t* + *dt*) owing to the state transition of node *i* is

$$\begin{array}{c} {}[\beta_{P}\sum_{j=1}^{N}a_{ji}P_{j}\left(t\right)+\beta_{D}d_{i}\theta \left(t\right)][1-d_{i}\theta \left(t\right)]dt \end{array}$$ (9)

if *X*_*i*_(*t*) = 0, and this net profit is zero otherwise. So, the expected net profit in the time interval [*t*, *t* + *dt*) owing to the state transition of node *i* is

$$\begin{array}{c} {}[\beta_{P}\sum_{j=1}^{N}a_{ji}P_{j}\left(t\right)+\beta_{D}d_{i}\theta \left(t\right)][1-d_{i}\theta \left(t\right)][1-I_{i}\left(t\right)-P_{i}\left(t\right)-N_{i}\left(t\right)]dt. \end{array}$$ (10)

Hence, the expected net profit resulting from performing the DDP strategy *θ* is

$$\begin{array}{c} P\left(\theta \right)=\int_{0}^{T}\sum_{i=1}^{N}[\beta_{P}\sum_{j=1}^{N}a_{ji}P_{j}\left(t\right)+\beta_{D}d_{i}\theta \left(t\right)][1-d_{i}\theta \left(t\right)][1-I_{i}\left(t\right)-P_{i}\left(t\right)-N_{i}\left(t\right)]dt. \end{array}$$ (11)

Combining the above discussions, we may model the DDP problem as the following optimal control problem:

$$\begin{array}{c} \begin{array}{cc} & \max_{\theta \in \Theta }P\left(\theta \right)=\int_{0}^{T}F\left(x\left(t\right),\theta \left(t\right)\right)dt\\ & \;\text{s}.\text{t}.\;\frac{dx(t)}{dt}=f\left(x\left(t\right),\theta \left(t\right)\right),\;0\leq t\leq T,\\ & \;\;\;x(0)=0. \end{array} \end{array}$$ (12)

Here,

$$\begin{array}{c} F\left(x\left(t\right),\theta \left(t\right)\right)=\sum_{i=1}^{N}[\beta_{P}\sum_{j=1}^{N}a_{ji}P_{j}\left(t\right)+\beta_{D}d_{i}\theta \left(t\right)][1-d_{i}\theta \left(t\right)][1-I_{i}\left(t\right)-P_{i}\left(t\right)-N_{i}\left(t\right)]. \end{array}$$ (13)

We refer to this optimal control problem as a *DDP model*. In this model, each control represents a DDP strategy, the objective functional represents the expected net profit of the merchant under a DDP strategy, and an optimal control represents a DDP strategy that achieves the maximum possible expected net profit. The DDP model (12) is determined by the 9-tuple

$$\begin{array}{c} M_{DDP}=\left(G,T,\beta_{P},\beta_{D},\alpha_{P},\alpha_{N},\gamma_{P},\gamma_{I},\gamma_{N}\right). \end{array}$$ (14)

We refer to the problem of solving DDP models as the *DDP model problem*. In the subsequent section, we are going to develop a method for solving the DDP model problem by means of optimal control theory.

## 3 A method for solving the DDP model problem

This section is dedicated to developing a method for solving the DDP model problem. We proceed following this procedure: (1) prove the DDP model problem admits an optimal control, (2) derive the optimality system for solving the DDP model problem, and (3) describe an algorithm for numerically solving the DDP model problem.

### 3.1 The existence of an optimal control

Before starting out to solve the DDP model problem, we must first show that the problem is solvable, i.e., it admits an optimal control. To this end, we need the following lemma, which is a direct consequence of a well-known theorem in optimal control theory [36].

**Lemma 1**. *The DDP model* (12) *has an optimal control if the following six conditions hold simultaneously*.

*(C_1_)* Θ *is closed*.
*(C_2_)* Θ *is convex*.
*(C_3_) There exists θ* ∈ Θ *such that* $\frac{dx(t)}{dt}=f\left(x\left(t\right),\theta \left(t\right)\right)$ (0 ≤ *t* ≤ *T*) *is solvable*.
*(C_4_)* **f**(**x**, *θ*) *is bounded by a linear function in* **x**.
*(C_5_) F*(**x**, *θ*) *is concave on* Θ.
*(C_6_) There exist ρ* > 1, *c*_1_ > 0 *and c*_2_ *such that F*(**x**, *θ*) ≥ *c*_1_ ‖*θ*‖^*ρ*^ + *c*_2_.

**Remark 2**. *To help understand the lemma, below let us elaborate the roles of the six conditions involved in the lemma. First, it is obvious that a control is feasible if and only if it falls into* Θ *and makes the constraint system* (7) *solvable. Hence, the third condition formally states that the optimal control problem has a feasible control. This is the foundation for solving the model. Second, it follows from convexity analysis theory* [37] *that the second and fifth conditions imply that the objective functional is concave and hence is likely to have maximum as desired. Third, recall that the concave function* $f\left(x\right)=\frac{x}{1+x}$ *defined on the interval* [0, 1) *has no maximum, because its domain is not closed. Hence, the first condition is necessary for the objective functional to have maximum. Finally, it follows from optimal control theory that these three conditions together with the remaining two technical conditions indeed guarantee the existence of an optimal control*.

We are ready to show the existence of an optimal control.

**Theorem 1**. *The DDP model* (12) *admits an optimal control*.

*Proof*: Let θ* be a limit point of Θ. Then there exists a sequence of points, *θ*_1_, *θ*_2_, ⋯, in Θ that approaches θ*. As *L*[0, *T*]⋂*L*^2^[0, *T*] is complete, we have θ* ∈ *L*[0, *T*]⋂*L*^2^[0, *T*]. Hence, the closeness of Θ follows from the observation that 0 ≤ θ* = lim_*n*→∞_ *θ*_*n*_ ≤ 1.

Let *θ*_1_, *θ*_2_ ∈ Θ, 0 < *η* < 1. As *L*[0, *T*]⋂*L*^2^[0, *T*] is a real vector space, we have (1 − *η*)*θ*_1_ + *ηθ*_2_ ∈ *L*[0, *T*]⋂*L*^2^[0, *T*]. Hence, the convexity of Θ follows from the observation that 0 ≤ (1 − *η*)*θ*_1_+ *ηθ*_2_ ≤ 1.

Let *θ* = 0. As **f**(**x**, 0) is continuously differentiable, it follows by Continuation Theorem for Differential Systems [38] that the differential system $\frac{dx(t)}{dt}=f\left(x\left(t\right),0\right)$ (0 ≤ *t* ≤ *T*) is solvable.

The fourth condition in Lemma 1 follows from the boundedness of **x** and *θ*. The concavity of *F*(**x**, *θ*) on Θ is obvious. Finally, we have *F*(**x**, *θ*) ≥ 0 ≥ *θ*^2^ − 1. It follows from Lemma 1 that the claim holds.

**Remark 3**. *Theorem 1 lays a solid foundation for solving the DDP model problem*.

### 3.2 The optimality system for the DDP model problem

The Hamiltonian of the DDP model (12) is

$$\begin{array}{c} \begin{array}{cc} & H(x(t),\theta (t),\lambda (t))\\ & =\sum_{i=1}^{N}[\beta_{P}\sum_{j=1}^{N}a_{ji}P_{j}\left(t\right)+\beta_{D}d_{i}\theta \left(t\right)][1-d_{i}\theta \left(t\right)][1-I_{i}\left(t\right)-P_{i}\left(t\right)-N_{i}\left(t\right)]\\ & +\sum_{i=1}^{N}\lambda_{i}\left(t\right)\{[\beta_{P}\sum_{j=1}^{N}a_{ji}P_{j}\left(t\right)+\beta_{D}d_{i}\theta \left(t\right)][1-I_{i}\left(t\right)-P_{i}\left(t\right)-N_{i}\left(t\right)]\\ & -\left(\alpha_{P}+\alpha_{N}+\gamma_{I}\right)I_{i}\left(t)\right\}+\sum_{i=1}^{N}\mu_{i}\left(t\right)[(\alpha_{P}I_{i}\left(t\right)-\gamma_{P}P_{i}\left(t\right)]\\ & +\sum_{i=1}^{N}\nu_{i}\left(t\right)[\alpha_{N}I_{i}\left(t\right)-\gamma_{N}N_{i}\left(t\right)], \end{array} \end{array}$$ (15)

where λ = (λ_1_, ⋯, λ_*N*_, *μ*_1_, ⋯, *μ*_*N*_, *ν*_1_, ⋯, *ν*_*N*_) is the adjoint.

We give a necessary condition for the optimal control of a DDP model as follows.

**Theorem 2**. *Suppose θ is an optimal control for the DDP model* (12), **x** *is the solution to the corresponding dynamical system* (7). *Then, there exists an adjoint λ such that*

$$\begin{array}{c} \left\{ \begin{array}{cc} \frac{d\lambda_{i}\left(t\right)}{dt} & =[\beta_{P}\sum_{j=1}^{N}a_{ji}P_{j}\left(t\right)+\beta_{D}d_{i}\theta \left(t\right)][1-d_{i}\theta \left(t\right)]+[\beta_{P}\sum_{j=1}^{N}a_{ji}P_{j}\left(t\right)+\beta_{D}d_{i}\theta \left(t\right)\\ & \;+\alpha_{P}+\alpha_{N}+\gamma_{I}]\lambda_{i}\left(t\right)-\alpha_{P}\mu_{i}\left(t\right)-\alpha_{N}\nu_{i}\left(t\right),\\ \frac{d\mu_{i}\left(t\right)}{dt} & =[\beta_{P}\sum_{j=1}^{N}a_{ji}P_{j}\left(t\right)+\beta_{D}d_{i}\theta \left(t\right)][1-d_{i}\theta \left(t\right)]-\beta_{P}\sum_{j=1}^{N}a_{ij}[1-d_{j}\theta \left(t\right)][1-I_{j}\left(t\right)\\ & \;-P_{j}\left(t\right)-N_{j}\left(t\right)]+[\beta_{P}\sum_{j=1}^{N}a_{ji}P_{j}\left(t\right)+\beta_{D}d_{i}\theta \left(t\right)]\lambda_{i}\left(t\right)\\ & \;-\beta_{P}\sum_{j=1}^{N}a_{ij}[1-I_{j}\left(t\right)-P_{j}\left(t\right)-N_{j}\left(t\right)]\lambda_{j}\left(t\right)+\gamma_{P}\mu_{i}\left(t\right),\\ \frac{d\nu_{i}\left(t\right)}{dt} & =[\beta_{P}\sum_{j=1}^{N}a_{ji}P_{j}\left(t\right)+\beta_{D}d_{i}\theta \left(t\right)][1-d_{i}\theta \left(t\right)]+[\beta_{P}\sum_{j=1}^{N}a_{ji}P_{j}\left(t\right)+\beta_{D}d_{i}\theta \left(t\right)]\lambda_{i}\left(t\right)\\ & \;+\gamma_{N}\nu_{i}\left(t\right),\\ & \;0\leq t\leq T,1\leq i\leq N. \end{array}\right. \end{array}$$ (16)

*with* λ(*T*) = **0**. *Moreover*,

$$\begin{array}{c} \theta \left(t\right)=\max\{\min\{\frac{g(t)}{h(t)},1\},0\},\;0\leq t\leq T, \end{array}$$ (17)

*where*

$$\begin{array}{c} \begin{array}{cc} g(t) & =\sum_{i=1}^{N}d_{i}[1-I_{i}\left(t\right)-P_{i}\left(t\right)-N_{i}\left(t\right)][1+\lambda_{i}\left(t\right)-\frac{\beta_{P}}{\beta_{D}}\sum_{j=1}^{N}a_{ji}P_{j}\left(t\right)],\\ h(t) & =2\sum_{i=1}^{N}d_{i}^{2}[1-I_{i}\left(t\right)-P_{i}\left(t\right)-N_{i}\left(t\right)]. \end{array} \end{array}$$ (18)

*Proof*: It follows from Pontryagin Maximum Principle [39] that there exists λ such that

$$\begin{array}{c} \left\{ \begin{array}{cc} \frac{d\lambda_{i}\left(t\right)}{dt} & =-\frac{\partial H(x(t),\theta (t),\lambda (t))}{\partial I_{i}},\;0\leq t\leq T,1\leq i\leq N,\\ \frac{d\mu_{i}\left(t\right)}{dt} & =-\frac{\partial H(x(t),\theta (t),\lambda (t))}{\partial P_{i}},\;0\leq t\leq T,1\leq i\leq N,\\ \frac{d\nu_{i}\left(t\right)}{dt} & =-\frac{\partial H(x(t),\theta (t),\lambda (t))}{\partial N_{i}},\;0\leq t\leq T,1\leq i\leq N. \end{array}\right. \end{array}$$ (19)

Eq (16) follow by direct calculations. As the terminal cost is unspecified, and the final state is free, the transversality condition λ(*T*) = **0** holds. Again by Pontryagin Maximum Principle [39], we have

$$\begin{array}{c} H(x(t),\theta (t),\lambda (t))\in \arg\min_{\theta \in \Theta }H(x(t),\theta ,\lambda (t)),\;0\leq t\leq T. \end{array}$$ (20)

So,

$$\begin{array}{c} \begin{array}{cc} \theta (t) & \in \arg\min_{0\leq \theta \leq 1}\{\theta \sum_{i=1}^{N}d_{i}[1-I_{i}\left(t\right)-P_{i}\left(t\right)-N_{i}\left(t\right)][1+\lambda_{i}\left(t\right)-\frac{\beta_{P}}{\beta_{D}}\sum_{j=1}^{N}a_{ji}P_{j}\left(t\right)]\\ & \;-\theta^{2}\sum_{i=1}^{N}d_{i}^{2}[1-I_{i}\left(t\right)-P_{i}\left(t\right)-N_{i}\left(t\right)],0\leq t\leq T. \end{array} \end{array}$$ (21)

Eq (17) follows by direct calculations.

**Remark 4**. *Recall from the multivariate calculus theory* [40] *that to optimize a multivariate function subject to a set of equality constraints, we need to introduce a set of auxiliary parameters known as the Lagrange multipliers to incorporate the constraints into the objective function. As a result, the original constrained optimization problem boils down to an unconstrained optimization problem that is solvable relatively easily. Adjoints in optimal control theory are something like Lagrange multipliers in multivariate function optimization theory*.

By optimal control theory, the optimality system for the DDP model (12) consists of Eqs (7), (16) and (17), **x**(0) = **0**, and λ(*T*) = **0**. By solving the optimality system, we can get a unique DDP strategy. Theorem 1 guarantees that this DDP strategy is indeed an optimal DDP strategy. In the next subsection, we are going to present an algorithm for numerically solving optimality systems.

### 3.3 An algorithm for solving optimality systems

Inspired by the forward-backward sweep method for solving ordinary differential equations [41], in Algorithm 1 we describes an algorithm (the DDP algorithm) for numerically solving the optimality system of a DDP model, where ||*ϕ*|| = sup_0≤*t*≤*T*_|*ϕ*(*t*)|. In all of the following experiments, we set *ϵ* = 10^−6^, *K* = 10^3^. The DDP strategy obtained by running the DDP algorithm on a DDP model is a numerical version of the optimal DDP strategy. In the next section, we are going to solve some DDP models.

**Algorithm 1 DDP**

**Input**: DDP model $M_{DDP}=\left(G,T,\beta_{P},\beta_{D},\alpha_{P},\alpha_{N},\gamma_{P},\gamma_{I},\gamma_{N}\right)$, convergence error *ϵ*, upper bound *K* on the number of iterations.

**Output**: DDP strategy *θ*.

1: *θ*^(0)^ = **0**; *k* ≔ 0;

2: // generate the final DDP strategy through iterations; //

3: **repeat**

4:  *k* = *k* + 1;

5:  use the system (7) with *θ* = *θ*^(*k*−1)^ and **x**(0) = **0** to forwardly calculate **x**; **x**^(*k*)^ ≔ **x**;

6:  use the system (16) with *θ* = *θ*^(*k*−1)^, **x** = **x**^(*k*)^, and λ(*T*) = **0** to backwardly calculate λ; λ^(*k*)^: = λ;

7:  use the system (17) with **x** = **x**^(*k*)^ and λ = λ^(*k*)^ to calculate *θ*; *θ*^(*k*)^ = *θ*;

8: **until** ||*θ*^(*k*)^ − *θ*^(*k*−1)^|| < *ϵ* or *k* ≥ *K*

9: return *θ*^(*k*)^.

## 4 Examples of optimal DDP strategy

In this section, we execute the DDP algorithm given in the previous section on the corresponding DDP models to obtain the corresponding optimal DDP strategies.

### 4.1 Scale-free network

**Fig 2:**
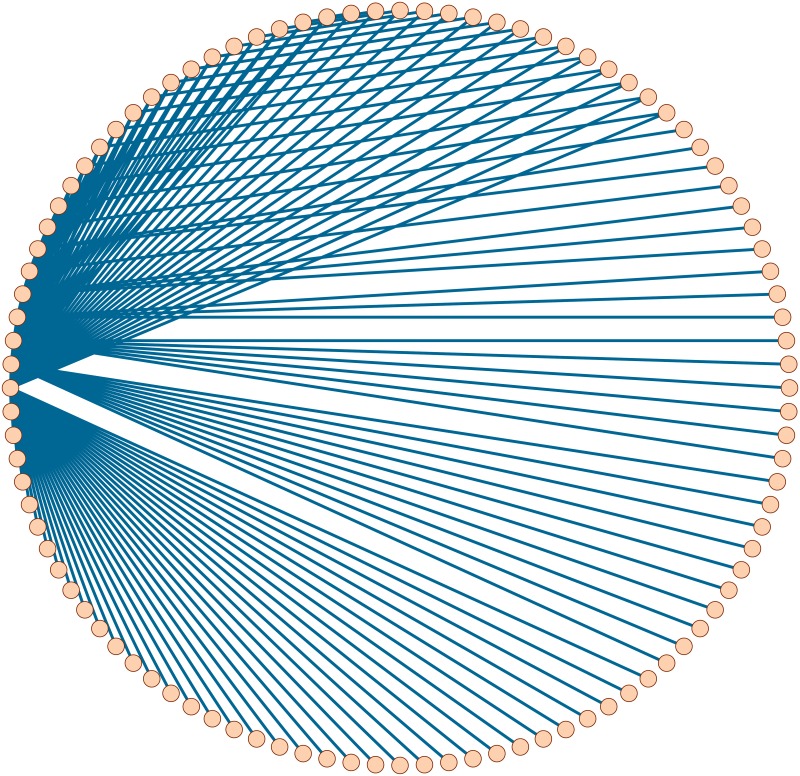
A synthetic scale-free network *G*_*SF*_.

Scale-free networks are networks with an approximate power-law degree distribution. It was reported that many real-world networks are scale-free [15, 16]. By using the Pajek software [42], we get a synthetic scale-free network *G*_*SF*_ on 100 nodes. See Fig 2.

**Fig 3:**
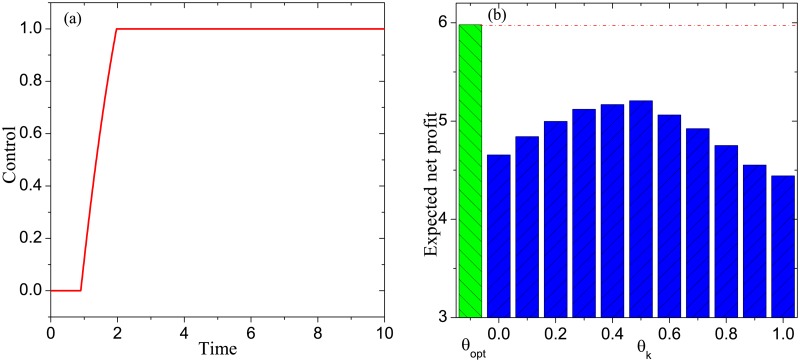
Experimental results in Example 1: (a) the optimal control, (b) the comparison of the optimal control with a set of static controls in terms of the expected net profit.

**Example 1**. *Consider the DDP model with G* = *G*_*SF*_, *T* = 10, *β*_*P*_ = 0.1, *β*_*D*_ = 0.1, *α*_*P*_ = 0.2, *α*_*N*_ = 0.1, *γ*_*P*_ = 0.3, *γ*_*I*_ = 0.2, *γ*_*N*_ = 0.1. *By solving the associated optimality system, we get an optimal control* *θ*_*opt*_, *which is shown in* Fig 3(a). *Define a set of static controls as follows*: *θ*_*k*_ = 0.1 × *k*, *k* = 0, 1, ⋯, 10. Fig 3(b) *exhibits* P(θ), *θ* ∈ {*θ*_*opt*_}⋃{*θ*_*k*_: *k* = 0, 1, ⋯, 10}. *It is seen that the optimal control is superior to all of the remaining controls in terms of the expected net profit*.

### 4.2 Small-world network

**Fig 4:**
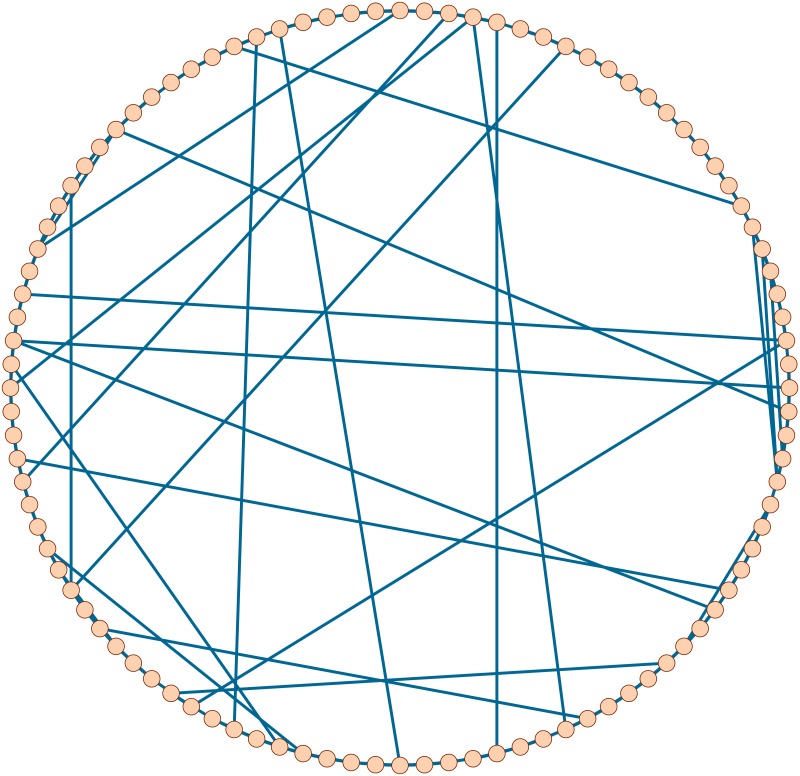
A synthetic small-world network *G*_*SW*_.

Small-world networks are networks with a relatively small diameter. It was reported that many real-world networks are small-world [14, 16]. By using Pajek, we get a synthetic small-world network *G*_*SW*_ on 100 nodes. See Fig 4.

**Fig 5:**
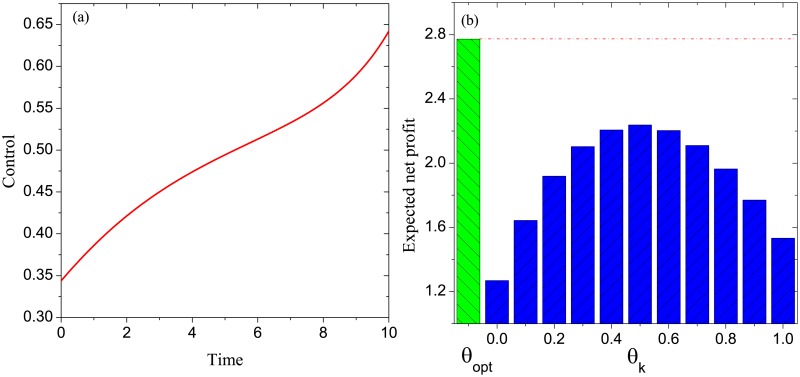
Experimental results in Example 2: (a) the optimal control, (b) the comparison of the optimal control with a set of static controls in terms of the expected net profit.

**Example 2**. *Consider the DDP model with G* = *G*_*SW*_, *T* = 10, *β*_*P*_ = 0.1, *β*_*D*_ = 0.2, *α*_*P*_ = 0.1, *α*_*N*_ = 0.1, *γ*_*P*_ = 0.3, *γ*_*I*_ = 0.2, *γ*_*N*_ = 0.1. *By solving the associated optimality system, we get an optimal control θ*_*opt*_, *which is shown in* Fig 5(a). *Define a set of static controls as follows*: *θ*_*k*_ = 0.1 × *k*, *k* = 0, 1, ⋯, 10. Fig 5(b) exhibits P(θ), *θ* ∈ {*θ*_*opt*_}⋃{*θ*_*k*_: *k* = 0, 1, ⋯, 10}. *It is seen that the optimal control outperforms all of the remaining controls in terms of the expected net profit*.

### 4.3 Email network

**Fig 6:**
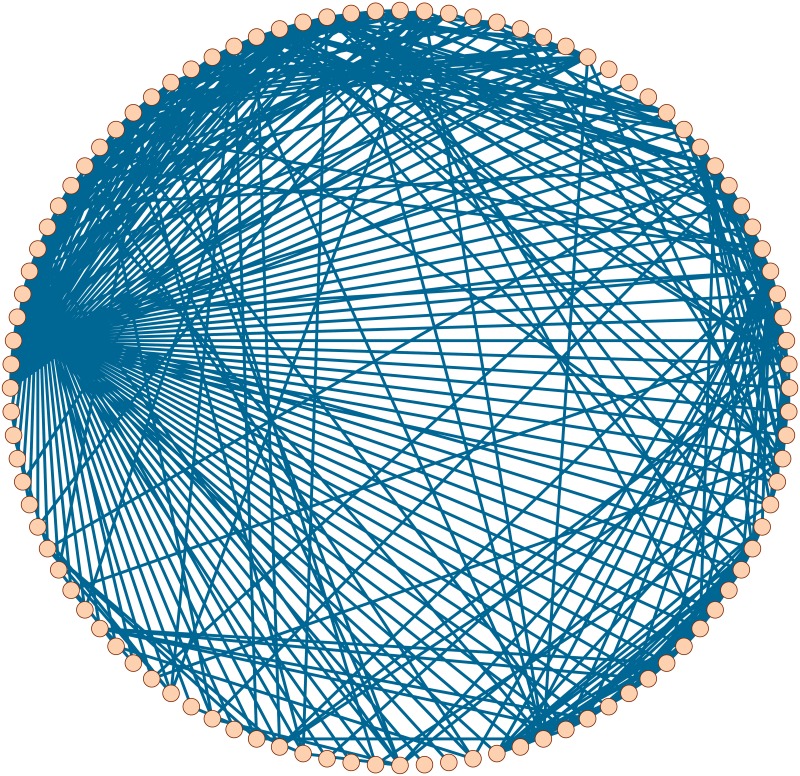
A realistic email network *G*_*EM*_.

Fig 6 exhibits a realistic email network *G*_*EM*_ on 100 nodes [43].

**Fig 7:**
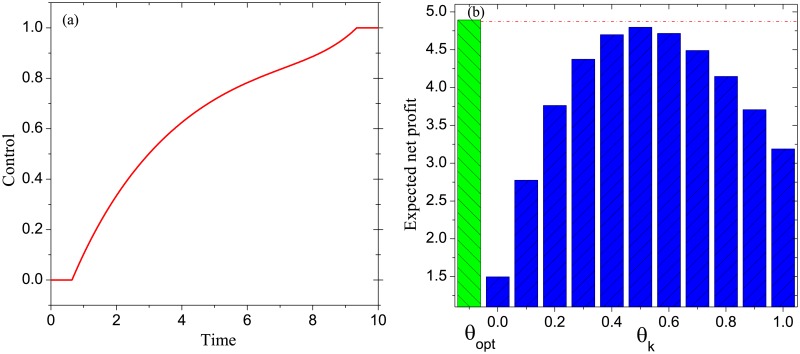
Experimental results in Example 3: (a) the optimal control, (b) the comparison of the optimal control with a set of static controls in terms of the expected net profit.

**Example 3**. *Consider the DDP model with G* = *G*_*EM*_, *T* = 10, *β*_*P*_ = 0.2, *β*_*D*_ = 0.1, *α*_*P*_ = 0.1, *α*_*N*_ = 0.2, *γ*_*P*_ = 0.3, *γ*_*I*_ = 0.2, *γ*_*N*_ = 0.1. *By solving the associated optimality system, we get an optimal control θ*_*opt*_, *hich is shown in* Fig 7(a). *Define a set of static controls as follows*: *θ*_*k*_ = 0.1 × *k*, *k* = 0, 1, ⋯, 10. Fig 7(b) *exhibits* P(θ), *θ* ∈ {*θ*_*opt*_}⋃{*θ*_*k*_: *k* = 0, 1, ⋯, 10}. *It is seen that the optimal control overmatches all of the remaining controls in terms of the expected net profit*.

By the above three experiments and 100 similar experiments, we conclude that for any DDP model, the following results hold:

- The optimal control is increasing over time. This conclusion tells us that, in practice, the basic discount rate should be enhanced gradually over time to gain the maximum possible marketing profit.
- The static control *θ*_*k*_ achieves the maximum expected net profit at *k* = 0.5. In practice, it may be infeasible to realize a dynamic basic discount rate. In this situation, the conclusion demonstrates that realizing the static basic discount rate of about 0.5 can achieve the maximum possible marketing profit.

## 5 The influence of some factors on the optimal expected net profit

In this section we examine the influence of some factors on the optimal expected net profit of a DDP model through computer experiments.

### 5.1 The two infection forces

First, we examine the influence of the two infection forces (positive infection force and discount infection force) on the optimal expected net profit.

**Fig 8:**
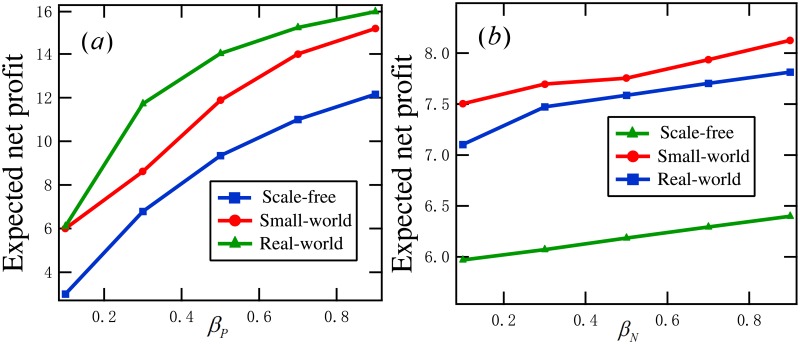
Experimental results in Example 4: (a) the optimal expected net profits for *β*_*D*_ = 0.4 and *β*_*P*_ ∈ {0.1, 0.3, ⋯, 0.9}, (b) the optimal expected net profits for *β*_*P*_ = 0.4 and *β*_*D*_ ∈ {0.1, 0.3, ⋯, 0.9}.

**Example 4**. *Consider a set of DDP models with T* = 10, *G* ∈ {*G*_*SF*_, *G*_*SM*_, *G*_*EM*_}, *α*_*P*_ = 0.2, *α*_*N*_ = 0.1, *γ*_*P*_ = 0.3, *γ*_*I*_ = 0.2, *γ*_*N*_ = 0.1. Fig 8(a) *plots* $P(\theta_{opt})$ *for* *β*_*D*_ = 0.4 and *β*_*P*_ ∈ {0.1, 0.3, ⋯, 0.9}. Fig 8(b) *displays* $P(\theta_{opt})$ *for* *β*_*P*_ = 0.4 and *β*_*D*_ ∈ {0.1, 0.3, ⋯, 0.9}. *rom this figure, we see that* $P(\theta_{opt})$ *is increasing with β*_*P*_ *and β*_*D*_, *respectively*.

Through this example and a set of 100 similar experiments, we conclude that the expected net profit of a DDP model is increasing with the positive infection rate and the discount infection rate, respectively. In practice, the merchant may enhance the discount infection rate by reducing the original prices of the relevant commodities. Generally, positive infection rate is not under the control of the merchant.

### 5.2 The two comment rates

Then, let us inspect the influence of the two comment rates (positive comment rate and negative comment rate) on the optimal expected net profit.

**Fig 9:**
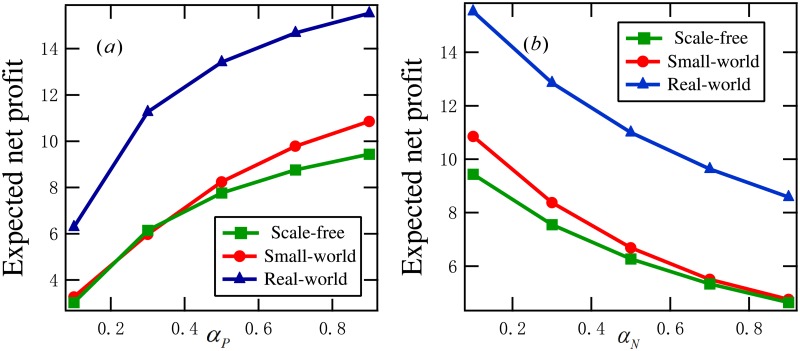
Experimental results in Example 5: (a) the optimal expected net profits for *α*_*N*_ = 0.1 and *α*_*P*_ ∈ {0.1, 0.3, ⋯, 0.9}, (b) the optimal expected net profits for *α*_*P*_ = 0.9 and *α*_*N*_ ∈ {0.1, 0.3, ⋯, 0.9}.

**Example 5**. *Consider a set of DDP models with T* = 10, *G* ∈ {*G*_*SF*_, *G*_*SM*_, *G*_*EM*_}, *β*_*P*_ = 0.2, *β*_*D*_ = 0.1, *γ*_*P*_ = 0.3, *γ*_*I*_ = 0.2, *γ*_*N*_ = 0.1. Fig 9(a) *exhibits* $P(\theta_{opt})$ *for* *α*_*N*_ = 0.1 *and α*_*P*_ ∈ {0.1, 0.3, ⋯, 0.9}. Fig 8(b) *shows* $P(\theta_{opt})$ *for* *α*_*P*_ = 0.9 *and* *α*_*N*_ ∈ {0.1, 0.3, ⋯, 0.9}. *From this figure, we see that* $P(\theta_{opt})$ *is increasing with α*_*P*_ *and decreasing with α*_*N*_, *respectively*.

From this example and a set of 100 similar experiments, we conclude that the expected net profit of a DDP model is increasing with the positive comment rate and decreasing with the negative comment rate, respectively. In practice, the merchant may enhance the positive comment rate and reduce the negative comment rate by enhancing the quality of the commodities or/and improving the user experience.

### 5.3 The three desire rates

Last, we examine the influence of the three desire rates (neutral desire rate, positive desire rate, and negative desire rate) on the optimal expected net profit.

**Fig 10:**
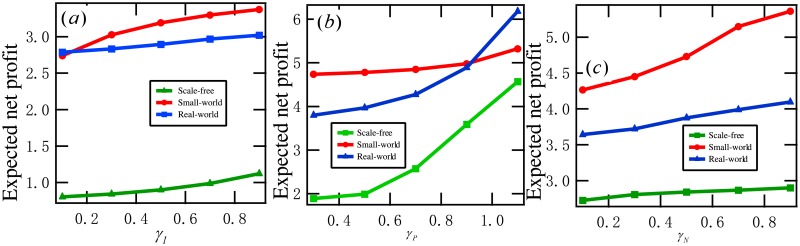
Experimental results in Example 6: (a) the optimal expected net profits for *γ*_*P*_ = 0.9, *γ*_*N*_ = 0.1, and *γ*_*I*_ ∈ {0.1, 0.3, ⋯, 0.9}, (b) the optimal expected net profits for *γ*_*I*_ = 0.3, *γ*_*N*_ = 0.1, and *γ*_*P*_ ∈ {0.3, 0.5, ⋯, 1.1}, (b) the optimal expected net profits for *γ*_*I*_ = 0.3, *γ*_*P*_ = 1.2, and *γ*_*N*_ ∈ {0.1, 0.3, ⋯, 0.9}.

**Example 6**. *Consider a set of DDP models with T* = 10, *G* ∈ {*G*_*SF*_, *G*_*SM*_, *G*_*EM*_}, *β*_*P*_ = 0.1, *β*_*D*_ = 0.2, *α*_*P*_ = 0.2, *α*_*N*_ = 0.1. Fig 10(a) *shows* $P(\theta_{opt})$ *for* *γ*_*P*_ = 0.9, *γ*_*N*_ = 0.1, and *γ*_*I*_ ∈ {0.1, 0.3, ⋯, 0.9}. Fig 10(b) plots $P(\theta_{opt})$ for *γ*_*I*_ = 0.3, *γ*_*N*_ = 0.1, *and* *γ*_*P*_ ∈ {0.3, 0.5, ⋯, 1.1}. Fig 10(c) *demonstrates* $P(\theta_{opt})$ *for γ*_*I*_ = 1, *γ*_*P*_ = 1.2, *and γ*_*N*_ ∈ {0.1, 0.3, ⋯, 0.9}. *From this figure, we see that* $P(\theta_{opt})$ *is increasing with γ*_*I*_, *γ*_*P*_, *and γ*_*N*_, *respectively*.

By this example and a set of 100 similar experiments, we conclude that the expected net profit of a DDP model is increasing with the neutral desire rate, the positive desire rate, and the negative desire rate, respectively. In practice, the merchant may enhance the three desire rates by improving the user experience.

## 6 Concluding remarks

This paper has studied the problem of developing cost-effective dynamic discount pricing strategies for viral marketing campaigns. We have modeled the problem as an optimal control problem and have solved it by means of optimal control theory.

Toward this direction, there are some open problems that are worth study. First, how to realize the recommended dynamic discount pricing strategies is a problem. Second, the influence index adopted in this paper may be replaced with some other influence measures [44–48] to improve the cost profit of the proposed dynamic discount pricing strategy. Third, the idea of this work may be applied to developing other kinds of viral marketing strategies. Next, this work may be extended to some other application scenarios such as malware containment [23–28], rumor restraint [29, 30, 49, 50], and cyber defense [31–33, 51]. Finally, a viral marketing campaign is essentially a game between the merchant and the customers, where the merchant goes after the maximum possible net profit, and the customers wish to buy the desired items at the lowest possible costs [52]. Therefore, it is expected that we can gain a deep insight into viral marketing through game-theoretic approach [33, 53–56].
